# Characteristics and Outcomes of Nonoperatively Managed Patients With Hip Fracture Using the Dutch Hip Fracture Audit

**DOI:** 10.1097/BOT.0000000000002778

**Published:** 2024-04-15

**Authors:** Hanne-Eva van Bremen, Lotta J. Seppala, Johannes H. Hegeman, Nathalie van der Velde, Hanna C. Willems, Hanne-Eva van Bremen, Lotta J. Seppala, Johannes H. Hegeman, Nathalie van der Velde, Hanna C. Willems

**Affiliations:** a Amsterdam Bone Center, Movement Sciences Amsterdam, Amsterdam, the Netherlands;; b Dutch Institute for Clinical Auditing, Leiden, the Netherlands;; c Amsterdam University Medical Centers, location Academic Medical Center, Internal Medicine and Geriatrics, University of Amsterdam, Amsterdam, the Netherlands;; d Amsterdam Public Health Research Institute, Amsterdam, the Netherlands; and; e Department of Trauma Surgery, Ziekenhuisgroep Twente, Almelo-Hengelo, the Netherlands.

**Keywords:** hip fracture, nonoperative management, audit

## Abstract

**OBJECTIVES:**

To identify and compare characteristics of patients with hip fracture treated nonoperatively versus those treated operatively.

**METHODS:**

***Design*:**

Retrospective cohort study.

***Setting*:**

Hip fracture population–based study.

***Patient Selection Criteria*:**

All adult patients with hip fractures (OTA/AO 31A and 31B) were included. Patients with pathological or periprosthetic hip fractures were excluded.

***Outcome Measures and Comparisons*:**

Patients were categorized according to the type of management (operative vs. nonoperative) and type of fracture (nondisplaced vs. other). Patient and fracture characteristics associated with nonoperative management (NOM) were analyzed.

**RESULTS:**

A total of 94,930 patients with hip fracture were included. Of these, 3.2% were treated nonoperatively. Patients receiving NOM were older [86 years (interquartile range, 79–91 years) vs. 81 years (interquartile range, 72–87 years); *P* < 0.001], more frequently institutionalized (42.4% vs. 17.6%), and were more dependent in activities of daily living (22.2% vs. 55.0%). Various clinical characteristics, including dementia [odds ratio (OR) 1.31 (95% confidence interval, CI, 1.18–1.45) *P* < 0.001], no functional mobility [OR 4.39 (95% CI, 3.14–3.68) *P* < 0.001], and activities of daily living (ADL) measured as KATZ-6-ADL [OR 1.17 (95% CI, 1.14–1.20) *P* < 0.001] were independently associated with NOM. Seven-day mortality was 37.6%, and 30-day mortality was 57.1% in patients treated nonoperatively.

**CONCLUSIONS:**

The first step in understanding patients who potentially benefit from NOM is evaluating the current standard of care. This study provides insight into the current hip fracture population treated nonoperatively. These patients are older, have higher percentage of dementia, more dependent, and show higher short-term mortality rates.

**LEVEL OF EVIDENCE:**

Therapeutic Level III. See Instructions for Authors for a complete description of levels of evidence.

## INTRODUCTION

Many patients with hip fracture do not live independently. Furthermore, high mortality rates highlight the frailty of this population. Hospital mortality is estimated to range from 3% to 7%, and 30-day mortality is reported up to 10%.^1,2^ Compared with the general population, a 3-fold increase in mortality is seen in patients with hip fracture.^3^ Patients with hip fracture with a high frailty score experience a 4-fold higher risk of 30-day mortality.^4^ General practice focuses on early surgical fixation to achieve pain relief and restore mobility. Despite surgical management, only 40%–60% of the patients regain their prefracture mobility state, and 40%–70% regain their prefracture independence level.^5–7^ Nonoperative management (NOM) is currently chosen in up to 10% of cases.^8–10^ However, which part of the hip fracture population may benefit from operative management is not known. In addition, little is known about the patient characteristics of patients selected for NOM.

The reason for NOM may vary depending on patient and fracture characteristics. NOM may be the best option for patients who are too unstable to undergo surgery or when the primary goal of treatment, regaining mobility, is not to be expected or when patients express a preference for NOM. In these cases, the focus shifts to maintaining quality of life and reducing pain. Currently, the Dutch guideline leaves no room for NOM for patients with a life expectancy of 6 weeks or more.^11^

Concerning fracture characteristics, current guidelines suggest NOM may be considered in limited cases for nondisplaced or impacted femoral neck fractures.^12–14^ Because these fractures are expected to be stable, safe mobilization can be achieved, potentially yielding equivalent outcomes without surgery.^15,16^ The Dutch guideline recommends operative management explicitly for these patients classified as American Society of Anesthesiologists 1–2.^12^ On the other hand, the National Institute for Health and Care Excellence guideline suggests that more research is needed.^13^ The American guideline presents multiple treatment options, commenting that current evidence is limited. Overall, NOM may be a viable option for a select subgroup depending on specific patient and fracture characteristics and the main goal of treatment.

The characteristics and outcomes of the population treated nonoperatively are not entirely understood. Therefore, the aim of this study was to compare patient and fracture characteristics and outcomes in patients with hip fracture treated nonoperatively versus operatively using Dutch guidelines, with a focus on identifying factors associated with NOM.

## MATERIALS AND METHODS

### Study Design, Setting, and Participants

A retrospective cohort study was conducted using a nationwide hip fracture database. The data were obtained from the Dutch Hip Fracture Audit (DHFA). This study was designed and reported following the Strengthening the Reporting of Observational Studies in Epidemiology guidelines.^17^ Adult patients diagnosed with a femoral neck or intertrochanteric fracture (OTA/AO 31A and 31B12) between January 1, 2016, and January 1, 2023, in the Netherlands were included.^18^ Patients with a periprosthetic hip fracture or pathological hip fracture were not included. The DHFA is a nationwide registration that includes patients with hip fracture in the Netherlands undergoing operative or NOM. More than 95% of all hospitals in the Netherlands are included in the DHFA, resulting in a coverage of more than 85% of all patients with hip fracture.^19^ Hospitals provide data through a secure survey system completed by a clinician or batch data processing. Data processing has several built-in validation processes, and previous data verification has shown high completeness and accuracy of the data.^19^

### Variables

Data on discharge destination, mobility at the time of discharge, length of hospital stay, and all-cause mortality at 7 days, 30 days, 3 months, and 1 year were extracted from the database. All-cause mortality was stratified based on nondisplaced femoral neck fractures and other types of fractures and measured in patients included between 2016 and 2021 to allow the measurement of 3-month and 1-year mortality. Dates of death were calculated using the data from health insurance reimbursements by a third party using social security numbers. Therefore, patients were excluded from further mortality analysis if the social security number was unknown. Mobility at the time of discharge was available up until 2021.

Data on age, gender, fracture type, prefracture mobility score, dementia, osteoporosis, activities of daily living (ADL) score, risk of malnutrition, and prehospital living situation were extracted from the database. The presence of dementia and osteoporosis was confirmed if diagnosed by a general practitioner or documented in the records of the treating hospital. ADL score was calculated using the KATZ-ADL score, previously validated for the Dutch language.^20^ The risk of malnutrition was classified as high, medium, or no risk according to the Short Nutritional Assessment Questionnaire or Malnutrition Universal Screening Tool.^21,22^ Mobility score was calculated using the fracture mobility score, previously validated in patients with hip fracture.^23^

### Statistical Methods

Categorical variables are shown as percentages and exact numbers. Continuous variables are shown as median and interquartile range (IQR). Baseline characteristics between the operative and nonoperative groups were tested for statistical significance using Pearson χ^2^ test or Mann–Whitney *U* test. Within the NOM patient group, characteristics of patients with a nondisplaced femoral neck fracture and patients with other types of fractures were compared using the same tests. Statistical significance was set at a *P* value of ≤0.05.

Missing data varied between the variables and was up to 20%. Multivariate imputations by chained equations were used to impute the missing data.^24^ It was assumed that variables were missing at random. The demographic variables and the outcome variable (type of treatment) were used as predictors for imputation of missing data. The Bayesian polytomous regression model was used for categorical variables in the imputation. Univariate logistic regression analysis was used for dichotomous variables and predictive mean matching for continuous variables. A total of 20 imputed data sets were created.

Multivariable regression analysis was performed to investigate the potential associations between patient and fracture characteristics and NOM. For the selection of variables used in the multivariable logistic regression, a univariable logistic regression was performed. Variables were selected for multivariable regression analysis if the *P* value was less than 0.05. All variables included in the multivariable regression were adjusted for the other. The odds ratio (OR) and 95% confidence interval (95% CI) were reported.

Statistical analysis was performed using R Studio Version 4.2.1, using the “Dplyr,” “TableOne,” and “ggplot2” packages. The “Mice” package was used for multiple imputations and logistic regression.

## RESULTS

### Group Comparison

#### Operatively Versus NonOperatively Managed Patients with Hip Fracture

In total, 94,390 patients with a hip fracture were identified in the DHFA database, of whom 3065 (3.2%) received NOM and 91,865 (96.8%) received operative management (Table 1). Overall, all basic characteristics differed between the 2 groups, except gender. Compared with the operative group, patients receiving NOM were older [86 years (IQR 79–91 years) vs. 81 years (IQR 72–87 years), respectively; *P* < 0.001] and less mobile (23.8% only mobile indoors vs. 7.2%, respectively; *P* < 0.001). Before sustaining a fracture, the nonoperative group was more frequently institutionalized (47.2% vs. 19.6% *P* < 0.001) and was more dependent in ADL (KATZ-6 ADL >0: 60.8% vs. 39.9% *P* < 0.001).

**TABLE 1.** Clinical and Fracture Characteristics of Patients With Proximal Femur Fracture Treated Operatively and NonOperatively

Patient and Fracture Characteristics^a^	Nonoperative	Operative
Total number of patients	3065	91,865
Age [median (IQR)], y***	86 (79–91)	81 (72–87)
Age stratified (%), y***		
<50	50 (1.6)	2442 (2.7)
51–60	81 (2.6)	5127 (5.6)
61–70	197 (6.4)	11,646 (12.7)
71–80	542 (17.7)	24,696 (26.9)
81–90	1315 (42.9)	35,435 (38.6)
91>	880 (28.7)	12,519 (13.6)
Female (%) (Not Significant)	2005 (65.4)	60,309 (65.6)
Fracture type (%)***		
Femoral neck, nondisplaced	885 (28.9)	15,747 (16.8)
Femoral neck, displaced	911 (29.7)	34,258 (37.3)
Trochanteric type AO-A1	344 (11.2)	11,564 (12.6)
Trochanteric type AO-A2	394 (12.9)	17,229 (18.8)
Trochanteric type AO-A3	107 (3.5)	5294 (5.8)
Subtrochanteric	96 (3.1)	3499 (3.8)
Unspecified	227 (7.4)	1310 (1.4)
Prefracture mobility (%)***		
Unknown	345 (11.3)	4464 (4.9)
Not using any mobility aid	600 (19.6)	43,341 (47.2)
Mobile outdoors using 1 mobility aid	140 (4.6)	5972 (6.5)
Mobile outdoors with 2 aids or frame	940 (30.7)	26,605 (29.0)
Mobile indoors but never outside without help of others	731 (23.8)	6657 (7.2)
No functional mobility (using lower extremities)	239 (7.8)	1579 (1.7)
Daily living dependency (%)***		
Independent (KATZ6-ADL = 0)	680 (22.2)	50,494 (55.0)
Prefracture living situation (%)***		
At home	776 (25.3)	50,077 (54.5)
At home with daily help	773 (25.2)	15,295 (16.6)
Institutionalized	1300 (42.4)	16,173 (17.6)
Dementia (%)***		
Yes	1140 (37.2)	13,963 (15.2)
Osteoporosis (%)**		
Yes	352 (11.5)	9206 (10.0)
Risk of malnutrition (%)***		
No risk of malnutrition	1747 (57.0)	74,454 (81.0)
Slight/medium risk of malnutrition	159 (5.2)	3307 (3.6)
High risk of malnutrition	501 (16.3)	8540 (9.3)

† Not Significant = *P* > 0.05, * = *P* < 0.05, **P < 0.01, ***P < 0.001.

In univariate regression analysis, age, sex, fracture type, prefracture mobility, daily living dependence, prefracture living situation, dementia, osteoporosis, and risk of malnutrition were associated with NOM apart from gender [OR 0.99 (95% CI, 0.92–1.07)] (Table 2). In multivariate regression analysis, all variables apart from mobile outdoor using 1 mobility aid [OR 0.97 (95% CI, 0.79–1.18)], home with daily help [OR 1.06 (95% CI, 0.96–1.18)], and osteoporosis [OR 0.95 (95% CI, 0.84–1.07)] were independently associated with NOM. Low prefracture mobility scores, such as mobile indoor [OR 2.97 (95% CI, 2.59–3.40)] and no functional mobility [OR 4.39 (95% CI, 3.67–5.26)], dementia [OR 1.31 (95% CI, 1.18–1.45)], and medium and high risk of malnutrition [OR 1.51 (95% CI, 1.28–1.78), OR 1.73 (95% CI, 1.56–1.91)], were independently associated with NOM.

**TABLE 2.** Clinical Characteristics Associated With Nonoperative Management

Clinical Characteristics	Univariate Analysis		Multivariate Analysis	
	OR (95% CI)	*P*	OR (95% CI)	*P*
Age	1.05 (1.04–1.05)	<0.001	1.02 (1.02–1.03)	<0.001
Sex (female)	0.99 (0.92–1.07)	0.84		
Fracture type (femoral neck, nondisplaced)				
Femoral neck, dislocated	0.46 (0.42–0.51)	<0.001	0.37 (0.34–0.41)	<0.001
Trochanteric type AO-A1	0.52 (0.46–0.59)	<0.001	0.38 (0.33–0.43)	<0.001
Trochanteric type AO-A2	0.40 (0.35–0.45)	<0.001	0.27 (0.24–0.31)	<0.001
Trochanteric type AO-A3	0.35 (0.29–0.43)	<0.001	0.26 (0.21–0.33)	<0.001
Subtrochanteric	0.48 (0.39–0.59)	<0.001	0.40 (0.32–0.50)	<0.001
Not specified	3.03 (2.59–3.54)	<0.001	2.74 (2.31–3.26)	<0.001
Prefracture mobility (not using any mobility aid)				
Mobile outdoors using 1 mobility aid	1.68 (1.39–2.03)	<0.001	0.97 (0.79–1.18)	0.73
Mobile outdoors with 2 aids or frame	2.50 (2.27–2.76)	<0.001	1.24 (1.10–1.40)	<0.001
Mobile indoors but never outside without help of others	7.66 (6.88–8.53)	<0.001	2.97 (2.59–3.40)	<0.001
No functional mobility	10.66 (9.15–12.43)	<0.001	4.39 (3.67–5.26)	<0.001
Daily living dependency (KATZ6-ADL)	1.41 (1.38–1.43)	<0.001	1.17 (1.14–1.20)	<0.001
Prefracture living situation (institution)				
Home	0.20 (0.18–0.22)	<0.001	0.73 (0.64–0.84)	<0.001
Home with daily help	0.63 (0.58–0.69)	<0.001	1.06 (0.96–1.18)	0.51
Dementia (yes)	3.40 (3.14–3.68)	<0.001	1.31 (1.18–1.45)	<0.001
Osteoporosis (yes)	1.17 (1.05–1.31)	0.009	0.95 (0.84–1.07)	0.39
Risk of malnutrition (no risk)				
Slight/medium risk	2.07 (1.76–2.43)	<0.001	1.51 (1.28–1.78)	<0.001
High risk	2.52 (2.29–2.78)	<0.001	1.73 (1.56–1.91)	<0.001

#### Nondisplaced Femoral Neck Fractures Versus Other Fractures

Within the NOM group, 885 patients had a nondisplaced femoral neck fracture (Table 3). Compared with patients with other types of fractures, this group was younger, with a median age of 84 years (IQR 76–90 years; *P* < 0.001), had higher mobility scores before the fracture (28.2% not using any mobility aid vs. 16.3%, *P* < 0.001), and less independent in ADL (31.8% vs. 18.2%, *P* < 0.001). In the group with the nondisplaced femoral neck fracture, more patients lived independently at home (35.4%) compared with those with displaced fractures where a larger part of the patients were institutionalized 42.4% (*P* < 0.001).

**TABLE 3.** Baseline Characteristics Stratified to Fracture Type (Nondisplaced Proximal Femoral Neck Fracture and Other Types of Fractures)

Clinical characteristics†	Nonoperative	
	Femoral Neck, Nondisplaced	All Other Types
Total number of patients (n)	885	2079
Age [median, (IQR)], y***	84 (76–90)	87 (80–92)
Age stratified (%), y***		
<50	19 (2.1)	28 (1.3)
51–60	31 (3.5)	47 (2.7)
61–70	76 (8.6)	115 (5.5)
71–80	187 (21.1)	340 (16.4)
81–90	378 (42.7)	887 (42.7)
91>	194 (21.9)	662 (31.8)
Female (%) (Not Significant)	566 (64.0)	1376 (66.2)
Fracture type (%)		
Femoral neck, displaced	NA	911 (43.8)
Trochanteric type AO-A1	NA	344 (16.5)
Trochanteric type AO-A2	NA	394 (19.0)
Trochanteric type AO-A3	NA	107 (5.1)
Subtrochanteric	NA	96 (4.6)
Not specified	NA	222 (10.9)
Prefracture mobility (%)***		
Not using any mobility aid	250 (28.2)	339 (16.3)
Mobile outdoors using 1 mobility aid	48 (5.4)	86 (4.1)
Mobile outdoors with 2 aids or frame	260 (29.4)	659 (31.7)
Mobile indoors but never outside without help of others	156 (17.6)	559 (26.9)
No functional mobility (using lower extremities)	72 (8.1)	157 (7.6)
Daily living dependency (%)***		
Independent (KATZ6-ADL = 0)	281 (31.8)	378 (18.2)
Prefracture living situation (%)***		
At home	313 (35.4)	449 (21.6)
At home with daily help	215 (24.3)	543 (26.1)
Institutionalized	315 (35.6)	950 (45.7)
Dementia (%)***		
Yes	274 (31.0)	842 (40.5)
Osteoporosis (%)*		
Yes	85 (9.6)	261 (12.6)
Risk of malnutrition (%)***		
No risk of malnutrition	546 (61.7)	1143 (55.0)
Slight/medium risk of malnutrition	44 (5.0)	109 (5.2)
High risk of malnutrition	113 (12.8)	371 (17.8)

† Not Significant = *P* > 0.05, * = *P* < 0.05, **P < 0.01, ***P < 0.001.

NA, not applicable.

## PATIENT OUTCOMES

Table 4 provides an overview of the outcome measures of all nonoperative patients. Most patients were discharged to an institution (57.4%) and 43.8% had no functional mobility at discharge. The median hospital stay was 1 day (IQR 1–4 days). All-cause mortality was 36.4% in the first 7 days and increased to 56.9% at 30-day follow-up (Table 5). Three months and 1-year mortality was 62.8% and 68.8%, respectively. All-cause mortality rates at 7 and 30 days were 23.3% and 38.6% (*P* < 0.001) for nondisplaced femoral neck fractures and were higher than those seen for other fracture types, 46.6% and 69.1% (*P* < 0.001).

**TABLE 4.** Clinical Outcome Measures in Nonoperatively Treated Patients with Hip Fracture

	Total
Discharge destination (%)	N = 3065
Home	318 (10.4)
Home with daily help	250 (8.2)
Institutionalized	1758 (57.4)
Missing	739 (24.1)
Mobility at time of discharge (%)	N = 2226
Unknown	189 (8.5)
Not using any mobility aid	26 (1.1)
Mobile outdoors using 1 mobility aid	48 (2.2)
Mobile outdoors with 2 aids or frame	289 (12.3)
Mobile indoors but never outside without help of others	105 (4.7)
No functional mobility (using lower extremities)	976 (43.3)
Missing	593 (26.6)

**TABLE 5.** All-Cause Mortality in Nonoperatively Treated Patients with Hip Fracture

	Total (n = 2062)	Femoral Neck, Non-Displaced (n = 623)	All Other Types (n = 1282)
All-cause mortality (%)			
7 d	776 (37.6)	145 (23.3)	598 (46.6)
30 d	1178 (57.1)	241 (38.7)	887 (69.2)
3 mo	1301 (63.1)	276 (44.3)	966 (75.4)
1 y	1441 (69.8)	317 (50.9)	1048 (81.7)

The subgroup of patients with a nondisplaced femoral neck fracture alive at 90 days was younger [81 years (IQR 72–87 years) vs. 87 years (IQR 82–91 years), *P* < 0.001], less dependent (30.5% mobile with an aid vs. 41%, *P* < 0.001), and more independent in terms of ADL (50.3% ADL independent vs. 13%, *P* < 0.001) in comparison to patients who died at 90 days (data not shown).

## DISCUSSION

This Dutch registry–based cohort study showed an overall incidence of NOM of 3.2%. Multivariate regression analysis showed that multiple clinical characteristics were independently associated with NOM. These patients were older, had poorer prefracture mobility, were more dependent on ADL, were more likely to come from a nursing home, and have dementia than those who underwent surgical treatment for their hip fracture. These results should be interpreted in light of the Dutch guideline stating the explicit advice to treat patients operatively with a life expectancy of 6 weeks or more. The patient group treated nonoperatively with nondisplaced femoral neck fractures was relatively fitter in terms of mobility and ADL and had lower all-cause mortality than those with other types of fractures.

### Patient Characteristics and Outcomes

This study showed that age, prefracture living situation, mobility, KATZ-ADL, and dementia were associated with NOM. A systematic review reported percentages varying between 19% and 61% of dementia.^25^ This review included numerous studies with heterogeneous cohorts and potentially different reasons for NOM, which may lead to variation in percentages of dementia. One previous study in which clinical characteristics associated with NOM were identified used a comparable method to this study.^26^ After analyzing 1307 patients, the following factors were identified: age >90 years, sex, nursing home, and pertrochanteric fracture, similar to this study. Furthermore, this study identified higher dependence in ADL and higher risk of malnutrition in patients treated nonoperatively, which has not been described previously.

All-cause mortality in all patients treated nonoperatively in this study was high: 36.4% within 7 days and 62.8% within 90 days. The recommendations in the Dutch guideline that the population treated nonoperatively should have a life expectancy of <6 weeks likely explain the high mortality rate identified in this study. By contrast, previous studies reported lower 7-day, 3-month, and 1-year mortality rates (30 days: 15%–36.7%), which might be explained by the differences in the hip fracture population within the different studies.^24–28^ In previous studies, patients were younger, less institutionalized, and had a lower prevalence of dementia compared with this study. In the systematic review by Loggers et al, one-third of the patients were treated nonoperatively for nonmedical reasons, such as economic reasons or proxy preferences, which may contribute to the lower mortality rate. Variability in current guidelines regarding NOM, which range from the absence of recommendations to always considering surgical management, may lead to a variation in current practice.

#### Incidence

This study showed an overall prevalence of NOM of 3.2%, which is similar compared with the study by Johansen et al (1%–4%).^29^ By contrast, an Estonian population-based retrospective study from 2009 to 2017 reported a high incidence of 10%.^9^ However, no national guidelines were available for hip fracture treatment, which could explain the high incidence in Estonia at that time. In addition, in a follow-up study also performed by Prommik et al, a relatively high percentage of patients refused operative management (8%) or received NOM due to an expected good prognosis (15%).^30^ In 75.5% of the patients, NOM was the chosen type due to a poor expected prognosis.

This study may be subject to underregistration of nonoperatively treated hip fractures due to registration bias, leading to a lower reported prevalence. Because this study incorporated patients from 2016 to include a large number of patients, it is worth noting that data quality has improved over time.^19^ As a result, the underregistration risk may be more present in the initial years. Therefore, the actual incidence of NOM is likely higher than presented in this study.

#### Fracture Characteristics

Patients who received NOM for nondisplaced femoral neck fractures had favorable characteristics compared with the patients with other types of fractures (femoral neck, dislocated, trochanteric type AO-A1, AO-A2, AO-A3, and subtrochanteric); they were younger, had better prefracture mobility, and were more independent. This fracture type is unique because it shares similar treatment goals for operative management and NOM in limited cases with comparable functional outcomes. The favorable patient characteristics in this specific group might suggest that the aim of NOM is not solely pain relief, supported by the lower percentage of mortality and patient characteristics at 90 days in this group but functional recovery with regaining mobility. Although current practice leans toward operative management for this patient group, the discussion remains centered around the choice of management when international guidelines are considered. The Dutch guideline leaves no room for NOM in this patient group, explicitly stating the need for operative management. However, this study showed that a percentage of patients with nondisplaced femoral neck fractures were treated nonoperatively despite the current guideline. The deviation from the current guideline might be due to international guidelines or past habits. The risk of NOM in nondisplaced femoral neck fractures is avascular necrosis, secondary displacement, and nonunion. A study by Amsellem et al showed a percentage of secondary displacement of 30%, meaning that 70% of the patients with a Garden I fracture were successfully treated nonoperatively.^15^ They identified various predictors for secondary displacement: dementia, institutionalization, multiple comorbidities, and a history of repeated falls, suggesting that vital patients with a nondisplaced femoral neck fracture in a subgroup might be treated nonoperatively with a similar outcome. This mirrors the patient group identified in this study with nondisplaced femoral neck fractures because the characteristics of frailty align with the subgroup of patients at risk of NOM complications in nondisplaced femoral neck fractures. In summary, this subgroup of patients has distinct characteristics that may influence the diversity in clinical decisions. This is reflected by the difference in outcomes, making it a distinctive type of fracture that should be taken into consideration when determining treatment and providing counseling.

This study has several strengths. First, this nationwide study included a high number of patients treated either operatively or nonoperatively. Second, the data quality was maintained for the date of death due to linking registry data to declaration data. Third, this registry is a comprehensive registration, including a wide variety of clinical characteristics. This includes ADL and risk of malnutrition, which have never been described before in this context. It is noteworthy that these variables are of substantial importance for patients.^31,32^ Moreover, risk of malnutrition is important because it is associated with higher mortality in patients with hip fracture and has been linked to lower rehabilitation efficiency and outcome.^33,34^

Because of the retrospective nature of this registry-based study, there are also several limitations. First, because of the retrospective design, it is impossible to add any variables that may be of interest, including follow-up data. Consequently, the reasons for NOM were not known in this study, leading to the inclusion of a heterogeneous population. This varied from fit patients undergoing nonoperative treatment to regain mobility, to frail patients with a limited life expectancy. Second, registry data are subject to registration bias due to the quality of the registration by the hospitals, which may influence the quality of the data in the registry. However, because of the registry's existence for several years and improvement in the data quality, the risk of registration bias might be limited. Third, cohort studies are subject to missing data; in this study, missing data were up to 20% in clinical characteristics and up to 26% in outcomes. However, because multiple imputations could be used, which minimized the expected selection bias usually caused by missing data.

Identifying patients for whom NOM might be a suitable choice remains complex for both patients and clinicians. To gain valuable insight into the complex hip fracture population who will benefit from NOM, the initial step involves understanding the standard of our care for a large number of patients. Future studies should assess and compare patient-related measurements, including follow-up assessments between patients treated nonoperatively and operatively. This may serve as a foundational basis for developing patient-centered treatment strategies and optimizing outcomes in the context of NOM.

In conclusion, this study is the first to provide a clear insight into the current hip fracture population treated nonoperatively in the Netherlands and identified various patient and fracture characteristics independently associated with NOM. Patients in the nonoperative group were older, more dependent, and showed high mortality rates at 30 and 90 days. By contrast, patients with a nondisplaced femoral neck fracture treated nonoperatively were younger, less dependent, and have lower mortality rates compared with patients with other types of fractures.
